# Evaluation of new antibiotic cocktails against contaminating bacteria found in allograft tissues

**DOI:** 10.1007/s10561-016-9581-6

**Published:** 2016-09-07

**Authors:** Agnese Serafini, Erika Riello, Diletta Trojan, Elisa Cogliati, Giorgio Palù, Riccardo Manganelli, Adolfo Paolin

**Affiliations:** 1 Department of Molecular Medicine, University of Padua, Padua, Italy; 2 Treviso Tissue Bank Foundation, Piazzale Ospedale 1, 31100 Treviso, Italy; 3 Francis Crick Institute, The Ridgeway, Mill Hill, London, NW7 1AA UK

**Keywords:** Antibiotic cocktail, Tissue banking, Bioburden, Microbiology, Decontamination protocol

## Abstract

Contamination of retrieved tissues is a major problem for allograft safety. Consequently, tissue banks have implemented decontamination protocols to eliminate microorganisms from tissues. Despite the widespread adoption of these protocols, few comprehensive studies validating such methods have been published. In this manuscript we compare the bactericidal activity of different antibiotic cocktails at different temperatures against a panel of bacterial species frequently isolated in allograft tissues collected at the Treviso Tissue Bank Foundation, a reference organization of the Veneto Region in Italy that was instituted to select, recover, process, store and distribute human tissues. We were able to identify at least two different formulations capable of killing most of the bacteria during prolonged incubation at 4 °C.

**Electronic supplementary material:**

The online version of this article (doi:10.1007/s10561-016-9581-6) contains supplementary material, which is available to authorized users.

## Introduction

Microbiological contamination of retrieved tissues is a very important topic and a critical aspect of allograft safety, especially when dealing with multi-tissue donors. Tissues retrieved from cadavers and living donors are frequently contaminated as a consequence of the retrieval and handling process, as well as the donor’s inherent bio-burden. Contaminated tissues may represent a potential hazard to recipients, and may only be implanted in the new host when proven to be efficiently decontaminated (Gottesdiener 1989; Eastlund 2006). The spectrum and frequency of bacterial contamination in tissues is very heterogeneous, mainly depending on tissue and donor type. Heart valves are usually more contaminated than musculoskeletal tissues (Ireland and Spelman 2005). In addition, it is well known that commensal bacteria (e.g. *Staphylococci*) and enteric bacteria are the most prevalent isolated organisms (van Kats et al. 2010). Despite measures to minimize contamination, such as the reduction of cadaver time (the time elapsed between death and start of retrieval) and the number of persons attending the retrieval, there is always a risk of bacterial contamination. Consequently, decontamination is a critical aspect, and tissue banks worldwide have established a wide variety of protocols to eliminate bacteria and fungi from isolated tissues (Steffen et al. 2015; Jashari et al. 2007; Heng et al. 2013). The common decontamination procedure usually includes a treatment with an antibiotic cocktail at 4 °C (Germain et al. 2010); however, the decontamination methods used in 17 European cardiovascular tissue banks were recently found to have large methodological differences (composition of cocktails, time and temperature of treatment), suggesting the need to validate and standardize the procedures (De By et al. 2012).

The aim of this study is to identify a new cocktail more efficient at low temperatures than the one currently used at Treviso Tissue Bank Foundation (FBTV), a reference organization of the Veneto Region in Italy instituted for the purpose of selecting, recovering, processing, storing and distributing musculoskeletal and cardiovascular tissues and amniotic membranes.

Recently, Pitt et al. (2014) compared the activity of different antibiotic cocktails used in tissue banks in the United Kingdom at different temperatures against bacteria commonly isolated from contaminated tissues. As expected, the efficacy of the cocktails increased with temperature; however, cocktails containing ciprofloxacin, gentamicin and imipenem were those whose efficacy was less affected by temperature with a good efficacy also at 4 °C, the temperature of choice to decontaminate allografts. Since the cocktail currently in use at FBTV does not include any of these drugs, we hypothesised that we could increase decontamination efficacy using a new cocktail including them. Based on this hypothesis, we formulated and tested new antibiotic combinations including ciprofloxacin, gentamicin and imipenem, against a panel of bacterial species frequently isolated in tissues collected by FBTV, comparing their activity with that of the cocktail currently in use.

## Methods

### Identification of bacteria contaminating allograft tissues

Following collection from living or cadaveric donors, tissues were decontaminated twice: initially upon retrieval and subsequently after processing. Each decontamination step entailed incubation for 24–48 h at 4 °C in RPMI containing ceftazidime 240 µg/ml (Fresenius-Kabi), lincomycin 120 µg/ml, polymyxin B 100 µg/ml (Biochrom) and vancomycin 50 µg/ml (Hospira). Samples for microbiological analyses were collected upon retrieval and after each decontamination step. Samples were cultivated using BD BACTEC Fluorescent Test Technology (BM BACTECTM plus aerobic/F and anaerobic/F culture vials); Soybean-casein digest broth was used in a qualitative procedure for aerobic/anaerobic culture and recovery of bacteria and yeast. If positive, bacteria were isolated and identified using standard procedures.

### Bacterial strains, standard media and growth condition

Table 1 shows the 34 strains belonging to 28 bacterial species used in this study and their source. The strains were routinely grown at 37 °C in a controlled atmosphere (Tab. S1) in Blood agar, Chocolate agar (*Haemophylus* and *Granulicatella* genus) or MacConckey (*Proteus*, in order to avoid swarming motility). Minimal inhibitory concentration (MIC) and minimal bactericidal concentration (MBC) estimations were performed in standard media used in clinical practice: Mueller–Hinton II Broth Cation adjusted (Thermofisher) supplemented with 2–5 % horse lysed blood when needed (*Streptococcus*, *Corynebacterium* and *Gemella* genera), Brucella Broth (Thermofisher) supplemented with 10 mg/l vitamin K, 5 mg/l hemin and 5 % horse lysed blood (anaerobes and *Granulicatella* genera). Antibiotic stock solutions for polymyxin B (Biochrom), meropenem (Fresenius Kabi Italia), ceftazidime (Teva) and vancomycin (Normon) were prepared in water and stored at −80 °C in 30–40 μl aliquots. For the other drugs, we used commercially available ready-to-use injectable solutions: ciprofloxacin (Ciproxin 2 mg/ml, Fresenius Kabi Italia), gentamicin sulphate (40 mg/ml, Fisiopharma) and lincomycin (300 mg/ml Pfizer).

**Table 1:** List of the strains used in this work

Bacterial species	Strain name	Source
*Achromobacter xilosoxidans denitrificans*	CIP 77.15T	PC
*Acinetobacter baumannii*	AS1	MVP
*Aerococcus viridans*	AS2	MVP
*Aeromonas hydrophyla*	AS3	MVP
*Bacteroides fragilis*	AS4	MVP
*Corynebacterium striatum*	AS5	MVP
*Enterococcus faecalis*	ATCC29212	ATCC
*Enterococcus faecalis*	ATCC51299	ATCC
*Escherichia coli*	ATCC25922	ATCC
*Gemella morbillorum*	CIP 81.10T	PC
*Granulicatella adiacens*	AS6	MVP
*Haemophilus parainfluenzae*	AS7	MVP
*Klebsiella pneumoniae*	ATCC700603	ATCC
*Klebsiella pneumoniae*	ATCC1706	ATCC
*Kocuria kristinae*	CIP 81.69T	PC
*Lactobacillus salivarius*	AS7	MVP
*Leuconostoc mesenteroides mesenteroides*	CIP 102388	PC
*Micrococcus luteus*	AS8	MVP
*Moraxella osloensis*	CIP 100025	PC
*Peptostreptococcus anaerobius*	CIP 104411T	PC
*Propionibacterium acnes*	AS9	MVP
*Proteus mirabilis*	AS10	MVP
*Proteus mirabilis*	AS11	MVP
*Staphylococcus aureus*	AS12 (MRSA)	MVP
*Staphylococcus aureus*	ATCC2913 (MSSA)	ATTC
*Staphylococcus epidermidis*	AS13	MVP
*Staphylococcus epidermidis*	AS14	MVP
*Staphylococcus hominis*	AS15	MVP
*Staphylococcus hominis*	AS16	MVP
*Staphylococcus hominis*	AS17	MVP
*Sphingomonas paucimobilis*	CIP 100752T	PC
*Streptococcus agalactiae*	AS18	MVP
*Streptococcus mitis*	AS19	MVP
*Streptococcus salivarius*	AS20	MVP

PC: Pasteur collection; MVP: microbiology and virology operating unit, Padua hospital agency; ATCC: collection

### Antimicrobial susceptibility

A 0.5 McFarland bacterial suspension (10^7^–10^8^ cells/ml) was diluted 1:100 in 11 ml of Mueller–Hinton II broth and aliquoted in a transparent 96-well plate (100 μl/well = 10^4^–10^5^ cells/well). In the first well, 100 μl of 2× antibiotic solution were added and then 1:2 scalar dilutions were performed. The final antibiotic concentration/well for each antibiotic is indicated in Table S2. As a consequence of the 1:2 dilutions, the final amount of cells was reduced to 10^3^–10^4^ cells/well. The 96-well plates were incubated at 37 °C until the appearance of a “pellet” at the bottom or cloudiness in the control well (without antibiotic). The minimum concentration able to inhibit growth (no pellet or cloudiness) was recorded as the MIC. Samples from the first three wells with no growth were spread on solid medium plates and incubated at 37 °C for 24–96 h (depending on the genus) to evaluate the MBC.

### Antibiotic cocktail formulations

The FBTV foundation currently uses a combination of 4 antibiotics, indicated in Table 2 as cocktail Z. Recently, Pitt et al. (2014) on behalf of National Health Service Blood and Transplant (NHSBT) compared bactericidal activity of several antibacterial and anti-fungal drugs combinations in bacterial and fungal strains commonly contaminating allograft tissues (some of which expressed significant levels of antibiotic resistance) at 4, 22 and at 37 °C. In this study, the cocktails with the highest efficacy at 4 °C were those ones containing gentamicin, vancomycin and imipenem or ciprofloxacin. On the basis of their data, we formulated 4 new cocktails (named A, B, C and D) containing different combinations of the antibiotics used by Pitt et al., but excluding the anti-fungal drugs (Table 2). Moreover, we replaced imipenem with meropenem due to its better availability and lower cost. We performed an initial preliminary screening test to evaluate the bactericidal activity of the test cocktails on 4 of the 34 selected bacterial strains (two Gram-positive and two Gram-negative strains with different MBC levels: *Escherichia coli*, *Proteus mirabilis* AS10, *Staphylococcus aureus* MSSA and *Staphylococcus hominis* AS15). The assay was performed by incubating bacteria in the cocktail for 24 or 48 h at 4 °C. Based on this preliminary experiment, we observed that cocktails A and B had lower activity than the others against *Staphylococcus* strains, and were therefore excluded from further analysis.

**Table 2:** Antibiotic cocktails used in this study

Antibiotic	Antibiotic cocktail (µg/ml)				
	Z	A	B	C	D
Ciprofloxacin	–	200	200	–	200
Ceftazidime	240	–	–	–	–
Gentamicin	–	200	–	200	200
Lyncomicin	120	–	–	–	–
Meropenem	–	200	200	200	–
Polymyxin B	100	–	–	–	
Vancomycin	50	100	100	100	100

### Cocktail evaluation

A 0.5 McFarland bacterial suspension (10^7^–10^8^ cells/ml) was diluted 1:100 (10^5^–10^6^ cells/ml) in 2.5 ml of BASE medium (Alchimia) to which 0.5 % of fetal calf serum and the different antibiotic cocktails were added. Samples were incubated at 22 or 4 °C without shaking. The number of CFU/ml was determined at different time points after adding the cocktail (T0, T6 h for samples incubated at 22 °C and T0, T24 h and T48 h for samples incubated at 4 °C). Viable counts (CFU/ml) were determined by plating 1:10 scalar dilutions of the bacterial suspension on solid media and counting the resulting colonies after incubation in the proper conditions. To avoid antibiotic carry-over, bacteria were pelleted at 10,000*g* for 5′ at 4 °C and suspended in the same volume of fresh medium before dilution. The bactericidal activity was calculated as:

$$\{ ({\text{CFU}}/{\text{ml}}\;{\text{after}}\;{\text{treatment}}) / ( {\text{CFU}}/{\text{ml}}\;{\text{before}}\;{\text{treatment)}}\} \times 100$$

## Results

### Bacterial species isolated from allograft tissues after decontamination

To select a panel of bacterial strains representing those currently contaminating tissues, we conducted a survey in 2012 and 2013 to isolate and identify all contaminating bacteria found in allografts at FBTV following the protocol actually used at FBTV reported in the Methods section. We singled out 102 bacterial species belonging to 47 genera (Tab. S3). Non-compliers species, according to FBTV policy (*Clostridium* spp., *Fungi/yeasts*, *Mycobacterium* spp., *Streptococcus pyogenes*, *Streptococcus pneumoniae*, *Pseudomonas aeruginosa*, *Serratia marcescens* and *Meningococcus* spp.), were excluded from the list. Species isolated in at least three different tissues (27 species belonging to 23 different genera), were selected for this study. For frequently detected species we decided to analyse more than one strain, making a total of 34 strains (Table 1).

### Assessment of antibiotic MBCs

To obtain a theoretical indication of the efficacy of the cocktails, we evaluated the MIC and MBC of each antibiotic for all strains (Table 3). Comparing the MBCs of the various strains, we made the following overall observations: (a) meropenem is the antibiotic with the greatest bactericidal effect, showing an MBC ≤1 µg/ml on 65 % of the strains, followed by ciprofloxacin with an MBC ≤2 µg/ml on 62 % of the strains; (b) meropenem and ciprofloxacin have a broad spectrum of action; (c) polymyxin B, ceftazidime and lincomycin showed the lowest bactericidal effect on most of the strains analysed, killing just 35, 53 and 39 % of the strains respectively, at a concentration of ≤8 µg/ml; (d) gentamicin resulted to be the best antibiotic or one the best for particularly low susceptible strains as *Klebsiella pneumoniae* ATCC1706, *Leuconostoc mesenteroides* and *Enterecoccus faecalis* ATCC29212.

**Table 3:** MIC and MBC

	Vancomycin		Meropenem		Ceftazidime		Ciprofloxacin		Polymyxin B		Lincomycin		Gentamicin	
	MIC	MBC	MIC	MBC	MIC	MBC	MIC	MBC	MIC	MBC	MIC	MBC	MIC	MBC
*Achromobacter xilosidans*	>32	>32	0.5	0.5	1	4	2	4	2	2	>128	>128	32	>32
*Acinetobacter baumannii*	>32	>32	≤0.25	≤0.25	8	8	0.125	0.5	4	4	>128	>128	1	1
*Aereococcus viridans*	0.125	0.5	0.25	0.25	1	>4	1	2	2	2	0.25	1	≤0.06	0.25
*Aeromonas hydrophila*	32	32	≤0.25	≤0.25	≤0.25	≤0.25	≤0.06	≤0.06	0.5	0.5	64	64	≤0.06	≤0.06
*Bacteroides fragilis*	8	16	≤0.25	0.5	>128	>128	4	>16	>64	>64	1	>4	32	>32
*Corynebacterium striatum*	0.25	0.25	0.5	1	128	>128	32	32	4	4	1	2	4	8
*Enterococcus faecalis ATCC29212*	2	4	4	4	64	128	0.5	1	64	64	8	>32	0.5	1
*Enterococcus faecalis ATCC 51299*	8	>32	16	128	>128	>128	0.5	8	>64	>64	>32	>32	>32	>32
*Escherichia coli*	>32	>32	≤0.25	≤0.25	≤0.25	0.5	≤0.06	0.25	1	1	>128	>128	0.5	0.5
*Gemella morbillorum*	0.5	>2	≤0.25	≤0.25	≤0.25	0.5	0.25	0.5	>64	>64	≤0.25	≤0.25	4	8
*Granulicatella adiacens*	1	1	≤0.25	≤0.25	1	>4	0.5	1	64	>64	1	>4	4	8
*Haemophilus parainfluenzae*	>32	>32	≤0.25	≤0.25	≤0.25	≤0.25	≤0.06	≤0.06	0.5	0.5	>128	>128	4	4
*Klebsiella pneumoniae* ATCC700603	>32	>32	≤0.25	≤0.25	32	32	0.25	0.5	2	2	>128	>128	4	4
*Klebsiella pneumoniae* ATCC1706	>32	>32	2	16	4	32	4	32	4	8	>128	>128	2	2
*Kocuria kristinae*	1	2	1	1	64	128	1	2	32	32	0.5	2	0.25	0.5
*Lactocobacillus salivarium*	32	>32	2	4	32	32	2	4	64	64	2	2	1	1
*Leuconostoc mesenteroides*	>32	>32	32	32	>128	>128	2	4	4	4	≤0.25	Nd	≤0.06	0.12
*Micrococcus luteus*	1	1	≤0.25	≤0.25	32	32	1	2	8	16	1	2	1	1
*Moraxella osloensis*	16	16	≤0.25	≤0.25	4	4	≤0.06	≤0.06	16	16	32	32	0.25	0.25
*Peptostreptococcus*	0.25	0.25	≤0.25	≤0.25	1	1	0.5	1	64	64	≤0.25	0.5	32	32
*Propionibacterium acnes*	0.5	0.5	≤0.25	0.5	1	4	0.5	0.5	32	64	≤0.25	>1	2	4
*Proteus mirabilis AS10*	>32	>32	≤0.25	≤0.25	8	8	1	2	>64	>64	>128	>128	32	>32
*Proteus mirabilis AS11*	>32	>32	≤0.25	2	≤0.25	8	≤0.06	0.125	>64	>64	>128	>128	4	4
*Sphingomonas paucimobilis*	8	32	≤0.25	≤0.25	4	8	1	2	32	64	>128	>128	≤0.06	0.12
*Staphylococcus aureus* MRSA	1	1	2	8	128	128	>32	>32	32	64	2	8	0.25	>1
*Staphylococcus aureus* MSSA	1	1	≤0.25	2	4	8	0.125	0.5	32	64	0.25	1	0.5	>2
*Staphylococcus epidermidis AS13*	2	4	16	>16	64	128	32	>32	32	64	>128	>128	16	>32
*Staphylococcus epidermidis AS14*	2	2	≤0.25	1	8	8	≤0.06	1	32	>64	1	16	≤0.06	0.06
*Staphylococcus hominis AS15*	1	>4	2	4	64	64	>32	>32	4	8	1	2	4	8
*Staphylococcus hominis AS16*	0.5	2	1	4	32	128	>32	>32	4	8	0.5	4	0.5	2
*Staphylococcus hominis AS17*	1	1	2	2	64	64	>32	>32	8	16	0.5	8	>32	32
*Streptococcus agalactiae*	2	>8	≤0.25	≤0.25	0.5	1	0.5	1	32	32	≤0.25	1	16	16
*Streptococcus mitis*	0.5	0.5	≤0.25	≤0.25	1	2	4	4	>64	>64	≤0.25	≤0.25	16	16
*Streptococcus salivarius*	0.5	>2	≤0.25	≤0.25	0.5	0.5	1	2	32	64	≤0.25	1	8	32

Antibiotic concentrations are indicated in µg/ml

### Efficacy of antibiotic cocktails at 4 °C

**Fig. 1:**
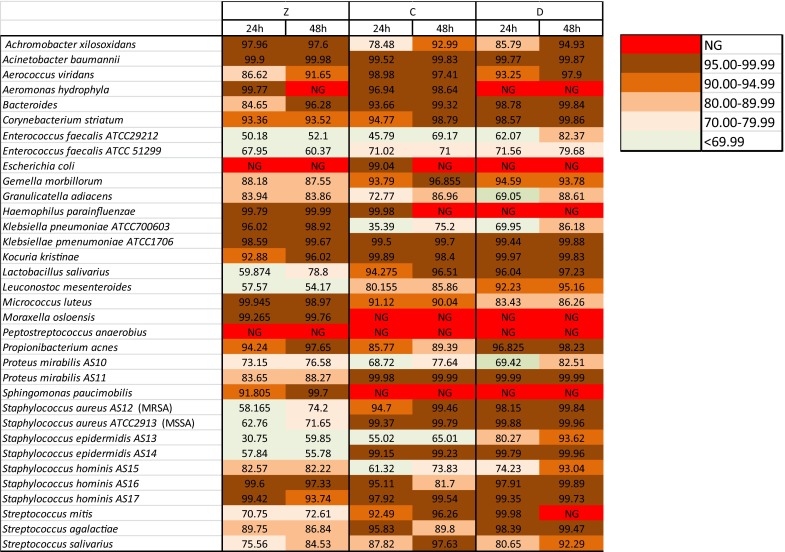
Bactericidal activity of antibiotic cocktails at 4 °C. Data shown as % kill; *NG* no growth

**Fig. 2:**
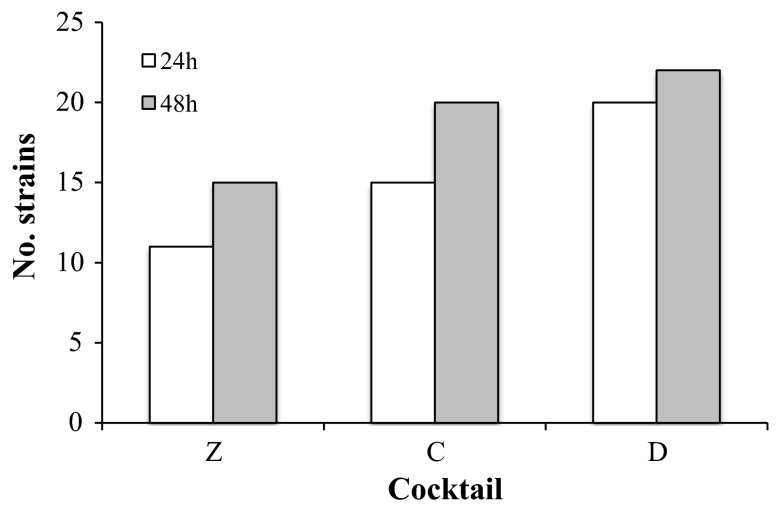
Effect of incubation time on bactericidal activity at 4 °C. The graph shows the number of strains killed at a tare higher than 95 % after treatment for 24 and 48 h with different antibiotic cocktails (Z, C and D)

**Fig. 3:**
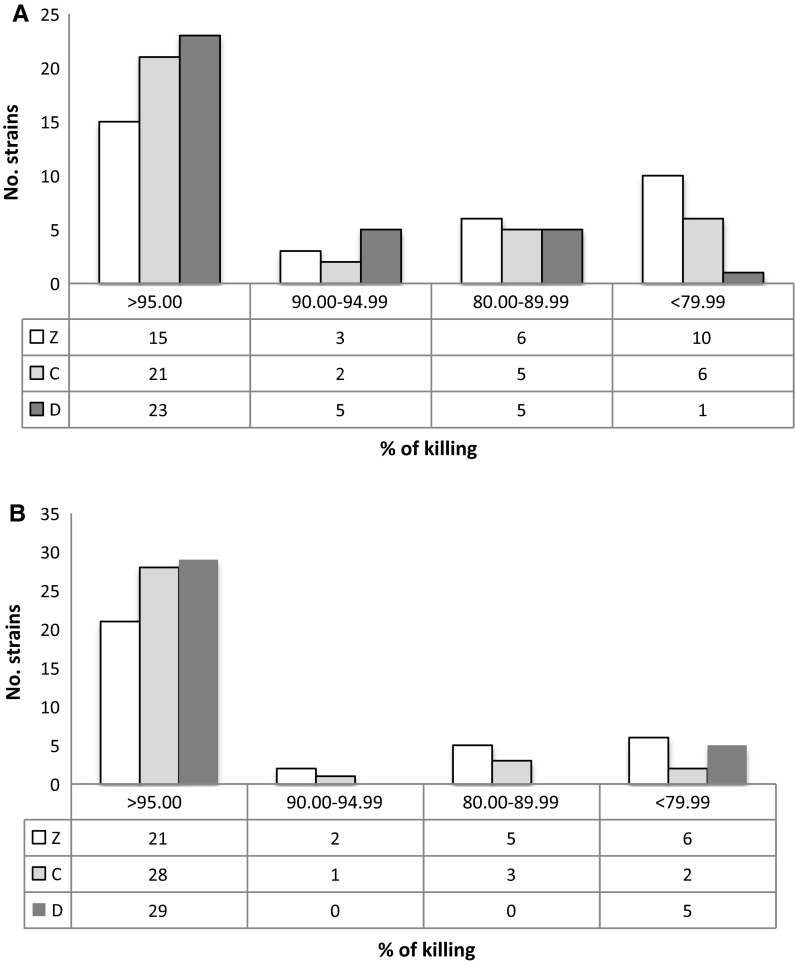
Effect of temperature on bactericidal activity. **a** Number of strains and different kill rates at 4 °C for 48 h. **b** Number of strains and different kill rates at 22 °C for 6 h

The efficacy of cocktails C and D at 4 °C was compared with that of the cocktail currently used at FBTV (cocktail Z). Figure 1 reports the bactericidal activity of cocktails Z, C and D for each strain, and clearly shows that the bactericidal activity of cocktails C and D was higher than that of cocktail Z on several bacterial strains, in particular against those belonging to *Streptococcus* and *Staphylococcus* genera. As expected, the activity increased with the extension of the treatment to 48 h (Figs. 1, 2). In particular, 21 out of 34 (61 %) and 23 out of 34 (67 %) total strains showed a kill rate higher than 95 % when incubated with cocktail C or D, respectively, compared to only 15 out of 34 (44 %) total strains for cocktail Z (Fig. 3a).

### Efficacy of antibiotic cocktails at 22 °C

**Fig. 4:**
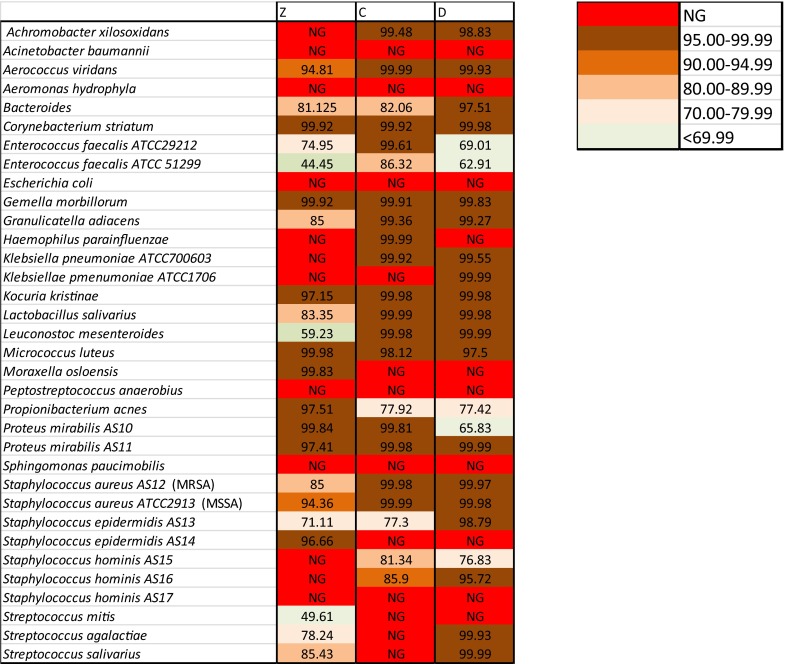
Bactericidal activity of antibiotic cocktails at 22 °C. Data shown as % kill; *NG* no growth

To evaluate the possibility of treating tissues at higher temperatures but for shorter periods, we tested the efficacy of these cocktails at 22 °C for 6 h. As expected, under such conditions the bactericidal activity of the cocktails was higher than at 4 °C, exceeding 95 % in most strains (Fig. 4). Again, cocktails C and D showed greater bactericidal activity: 28 out of 34 (82 %) and 29 out of 34 (85 %) total strains showed a kill rate higher than 95 % when incubated with cocktail C or D, respectively, compared to only 21 out of 34 (62 %) for cocktail Z (Fig. 3b).

## Discussion

Tissue decontamination is a challenge for tissue banks. However, the standardization and validation of specific decontamination procedures and antibiotic cocktails have rarely been pursued, consequently a wide range of antibiotic formulations, temperatures and exposure times are currently adopted for this purpose (Heng et al. 2013; De By et al. 2012; Germain et al. 2010).

Taking the assessments reported by Pitt et al. (2014), on the efficacy of different antibiotic cocktails at 4 and 22 °C against a wide panel of bacterial strains, we designed 4 different antibiotic cocktails. After a preliminary screening we focused on two of them which were tested against a panel of bacterial species commonly isolated from allograft tissues and compared them to the antibiotic cocktail currently in use in our facility at FBTV (cocktail Z). We did not include any anti-mycotic drugs, since in our procedure the presence of fungi in the pre-decontamination analysis is an exclusion criterion for tissue. For the same reason our bacterial panels did not include *Streptococcus pneumoniae*, *Streptococcus pyogenes*, *Serratia marcescens*, *Meningococcus* or *Pseudomonas aeruginosa* strains or strains belonging to the genus *Clostridium* or *Mycobacterium*.

The efficacy of the cocktails was tested both at 4 °C for 24 or 48 h and at 22 °C for 6 h. To prevent drug carry-over interfering with the results, the bacteria were rinsed in a drug-free medium before viability counts. Two of the new cocktails (C and D) killed a broad spectrum of bacteria even at 4 °C and were clearly more effective than cocktail Z. Specifically, after incubation for 48 h at 4 °C, 61 and 67 % of the strains showed a kill rate higher than 95 % when incubated with cocktail C or D, respectively, compared to 44 % of the strains reaching the same kill rate when incubated with cocktail Z. This effect was probably due to the broader spectrum of action of the antibiotics contained in the new cocktails compared to cocktail Z (Table 2). Cocktails C and D, both contained vancomycin and gentamicin, plus meropenem (cocktail C) or ciprofloxacin (cocktail D). The superiority of cocktails C and D over cocktail Z mainly lies in their increased bactericidal activity against *Streptococcus* and *Staphylococcus* strains (Fig. 1), which are the most commonly detected genera in allografts. In particular, cocktail D showed a kill rate higher than >90 % activity against all tested strains of *Streptococcus* and *Staphylococcus*.

Treatment at a higher temperature (such as room temperature, 22 °C) for shorter times would increase decontamination efficacy (in particular allowing higher depletion of more resistant species including *Enterecoccus faecalis*) and reduce processing times, however this procedure might not be applicable to all tissues due to their shorter survival at this temperature. It would be interesting in the future to measure tissue vitality at this temperature by performing specific tests, and to assess the efficacy of short decontamination procedures using shorter incubation times.

It is important to point out that susceptibility towards antibiotics varies within species, so the panel of clinical and reference isolates used in this work cannot cover all of the possible susceptibility spectra of bacterial contaminants found in the field. One drawback of our work was that our killing experiments were performed in liquid medium, in different conditions compared to those usually experienced by the bacteria during allograft decontamination. To alleviate this drawback, the medium was added with 0.5 % foetal calf serum to increase the concentration of proteins, which are obviously highly represented in tissues and can bind with antibiotics.

With regard to the above mentioned issue, we will shortly be analysing the microbiological results after one year with the new cocktail to verify its efficacy in allograft decontamination compared to cocktail Z.

It is also worth noting that our experiments were performed starting with cultures that had a bacterial concentration of 10^5^–10^6^ cells/ml, a much higher load than that found in allograft tissues, since a drug’s kill rate is usually inversely proportional to the bacterial load (i.e. inoculum effect; Udekwu et al. 2009); the bactericidal activity shown in Figs. 1 and 4 might be underestimated with respect to that obtained during allograft decontamination.

In conclusion, in this work we characterized two antibiotic formulations that displayed several advantages validating our initial hypothesis that a cocktail including gentamicin, ciprofloxacin or meropenem would be more efficient than the one currently in use at FBTV (cocktail Z): (1) both were found to be more potent both at 4 and at 22 °C; (2) both contained three antibiotics instead of four, reducing the cost of decontamination; and (3) both contained antibiotics which are more readily available than lyncomicin and polymyxin B (present in cocktail Z), as they are commonly used in clinical practice. In addition, in Italy polymixin B is no longer available as an antibiotic for systemic use, but only for topical use due to its toxicity.

## Electronic supplementary material

Below is the link to the electronic supplementary material.

Supplementary material 1 (DOCX 18 kb)

Supplementary material 2 (XLSX 13 kb)
