# Supplementary material for: Evaluation of new antibiotic cocktails against contaminating bacteria found in allograft tissues
Source: Cell Tissue Bank. 2016 Sep 7;17(4):619–28. doi: 10.1007/s10561-016-9581-6 (PMC5116045; doi:10.1007/s10561-016-9581-6)
Supplement: Supplementary file 1 — Supplementary material 1 (DOCX 18 kb) [file 10561_2016_9581_MOESM1_ESM.docx]

| Bacterial genus | Atmosphere^1^ |
| --- | --- |
| *Achromobacter* | NA |
| *Acinetobacter* | NA |
| *Aerococcus* | NA |
| *Aeromonas* | *NA* |
| *Bacteroides* | AA |
| *Corynebacterium* | CEA |
| *Enterococcus* | NA |
| *Escherichia* | NA |
| *Gemella* | CEA |
| *Granulicatella* | CEA |
| *Haemophilus* | CEA |
| *Klebsiella* | NA |
| *Kocuria* | NA |
| *Lactocobacillus* | NA |
| *Leuconostoc* | CEA |
| *Micrococcus* | CEA |
| *Moraxella* | NA |
| *Peptostreptococcus* | AA |
| *Propionobacterium* | AA |
| *Proteus* | NA |
| *Staphylococcus* | NA |
| *Sphingomonas* | NA |
| *Streptococcus* | CEA |

**Table S1**. Atmospheric compositions used in this study. NA: normal atmosphere; AA: anaerobic atmosphere: 90% N_2_, 5% H_2_, 5% CO_2_; CEA: carbon dioxide-enriched atmosphere: 5% CO_2_.

|  | Concentration (µg/ml) | | | | | | | | | |
| --- | --- | --- | --- | --- | --- | --- | --- | --- | --- | --- |
| Ceftazidime | 128 | 64 | 32 | 16 | 8 | 4 | 2 | 1 | 0.5 | 0.25 |
| Ciprofloxacin | 32 | 16 | 8 | 4 | 2 | 1 | 0.5 | 0.25 | 0.125 | 0.06 |
| Gentamicine | 32 | 16 | 8 | 4 | 2 | 1 | 0.5 | 0.25 | 0.125 | 0.06 |
| Lincomycin | 128 | 64 | 32 | 16 | 8 | 4 | 2 | 1 | 0.5 | 0.25 |
| Meropenem | 128 | 64 | 32 | 16 | 8 | 4 | 2 | 1 | 0.5 | 0.25 |
| Polymyxin B | 64 | 32 | 16 | 8 | 4 | 2 | 1 | 0.5 | 0.25 | 0.125 |
| Vancomycin | 32 | 16 | 8 | 4 | 2 | 1 | 0.5 | 0.25 | 0.125 | 0.06 |

**Table S2**. Range of antibiotic concentrations used for MIC and MBC evaluation.
